# Vancomycin-resistant enterococci (VRE) in hospital settings across European borders: a scoping review comparing the epidemiology in the Netherlands and Germany

**DOI:** 10.1186/s13756-023-01278-0

**Published:** 2023-08-12

**Authors:** Cansu Cimen, Matthijs S. Berends, Erik Bathoorn, Mariëtte Lokate, Andreas Voss, Alex W. Friedrich, Corinna Glasner, Axel Hamprecht

**Affiliations:** 1 Institute for Medical Microbiology and Virology, University of Oldenburg, Oldenburg, Germany; 2 Department of Medical Microbiology and Infection Prevention, University of Groningen, University Medical Center Groningen, Groningen, The Netherlands; 3 Department of Medical Epidemiology, Certe Medical Diagnostics and Advice Foundation, Groningen, The Netherlands; 4 University Hospital Muenster, University of Muenster, Muenster, Germany

**Keywords:** Vancomycin-resistant enterococci, VRE, Antibiotic resistance, Epidemiology, Prevalence, Dutch-German cross-border region, Germany, The Netherlands

## Abstract

The rising prevalence of vancomycin-resistant enterococci (VRE) is a matter of concern in hospital settings across Europe without a distinct geographical pattern. In this scoping review, we compared the epidemiology of vancomycin-resistant *Enterococcus* spp*.* in hospitals in the Netherlands and Germany, between 1991 and 2022. We searched PubMed and summarized the national antibiotic resistance surveillance data of the two countries. We included 46 studies and summarized national surveillance data from the NethMap in the Netherlands, the National Antimicrobial Resistance Surveillance database in Germany, and the EARS-Net data. In total, 12 studies were conducted in hospitals in the Netherlands, 32 were conducted in German hospitals, and an additional two studies were conducted in a cross-border setting. The most significant difference between the two countries was that studies in Germany showed an increasing trend in the prevalence of VRE in hospitals, and no such trend was observed in studies in the Netherlands. Furthermore, in both Dutch and German hospitals, it has been revealed that the molecular epidemiology of VREfm has shifted from a predominance of *vanA* towards *vanB* over the years. According to national surveillance reports, vancomycin resistance in *Enterococcus faecium* clinical isolates fluctuates below 1% in Dutch hospitals, whereas it follows an increasing trend in German hospitals (above 20%), as supported by individual studies. This review demonstrates that VRE is more frequently encountered in German than in Dutch hospitals and discusses the underlying factors for the difference in VRE occurrence in these two neighboring countries by comparing differences in healthcare systems, infection prevention control (IPC) guidelines, and antibiotic use in the Netherlands and Germany.

**Supplementary Information:**

The online version contains supplementary material available at 10.1186/s13756-023-01278-0.

## Background

Enterococci are among the most common nosocomial pathogens in the world [1]. The spread of multidrug-resistant enterococci in healthcare, the majority attributed to *Enterococcus faecium*, and their adaptation to the hospital environment have been of concern since the 1970s [1, 2].

Enterococci can acquire antibiotic resistance by sporadic chromosomal mutations or exogenous gene exchange, besides being intrinsically resistant to many antibiotics such as cephalosporins, trimethoprim-sulfamethoxazole, and lincosamides [3]. High-level resistance to aminoglycosides and resistance to ampicillin and glycopeptides are well-known examples of acquired antibiotic resistance in enterococci [3, 4]. The first case of vancomycin-resistant enterococci (VRE) was reported in France in 1986; since then, it has emerged as a major cause of nosocomial infections worldwide [5–7]. Vancomycin resistance has been attributed to the acquisition of gene clusters that alter the nature of peptidoglycan precursors; and to date, nine different gene clusters have been identified [8]: *vanA, vanB, vanC, vanD, vanE, vanG, vanL, vanM, vanN*. However, *vanA* and *vanB* are the major circulating gene clusters in human VRE colonization and infections, both in Europe and worldwide [5, 9].

Given the fact that VRE are resistant to first-line antibiotics in hospital settings, there are a limited number of therapeutic options, such as linezolid, tigecycline, and daptomycin [10]. However, increasing resistance to these last-resort antibiotics has been reported [10–14]. Therefore, prevention of VRE infections is crucial to avoid treatment challenges [15].

Over the past two decades, studies have provided information on the burden of VRE infections in hospitals [5, 16–20]. Compared to vancomycin-susceptible enterococci (VSE) infections, VRE infections are associated with higher morbidity, cost of care, longer length of hospital stay, and mortality [19, 21, 22]. Unsurprisingly, the World Health Organization (WHO) included VRE as a high-priority pathogen in its global list of important antibiotic-resistant bacteria in 2017 [23]. Data from the European Antimicrobial Resistance Surveillance Network (EARS-Net) justified the WHO’s decision by showing that the prevalence of VRE across Europe doubled from 2015 to 2019 [24]. According to this report, an increase in vancomycin resistance was reported across Europe due to the increasing prevalence of vancomycin-resistant *E. faecium* (VREfm) [24]. Interestingly, two neighboring countries, Germany and the Netherlands, are at both ends of the scale of the proportion of VREfm in all invasive *E. faecium* isolates according to EARS-Net (< 1% in the Netherlands and 22.3% in Germany). The underlying reasons for this difference are not yet fully understood [24]. Although Germany and the Netherlands have many common historical, cultural, and social values, they differ in many aspects regarding healthcare. These differences include amongst others the healthcare structure, antibiotic prescription habits, and local and national infection prevention and control (IPC) guidelines for multidrug-resistant microorganisms (MDRO) [25–27]. All these aspects taken together may be the cause for the differences in VRE rates encountered in these two neighboring countries [25, 27–29].

Despite the available evidence for the difference in the prevalence of VRE in the Netherlands and Germany, there are no nationwide comparative studies detailing this situation to date. Therefore, this review aims to describe the epidemiology of vancomycin-resistant *Enterococcus* spp. by presenting the outbreaks, VRE colonization prevalence, and VRE proportions in clinical isolates in hospitals in Germany and the Netherlands based on the literature and national and European surveillance data.

## Methods

We performed a scoping review using PubMed to search for publications in English, Dutch, and German providing data on VRE colonization and infection prevalence, incidence, surveillance, and outbreaks in hospital settings in the Netherlands and Germany. The review was performed following the recommendations of PRISMA-ScR [30]. We performed a peer-reviewed search strategy, executed on December 30, 2022. The search term (Additional file 1) was externally reviewed by a research librarian from the University of Groningen. The authors (CC and MSB) independently searched and extracted data using a peer-reviewed search strategy to avoid missing any relevant studies. No inconsistencies were encountered with this strategy. The dataset is available in Additional file 2, and those who are interested can reach out to the corresponding author for any further inquiries.

**Fig. 1:**
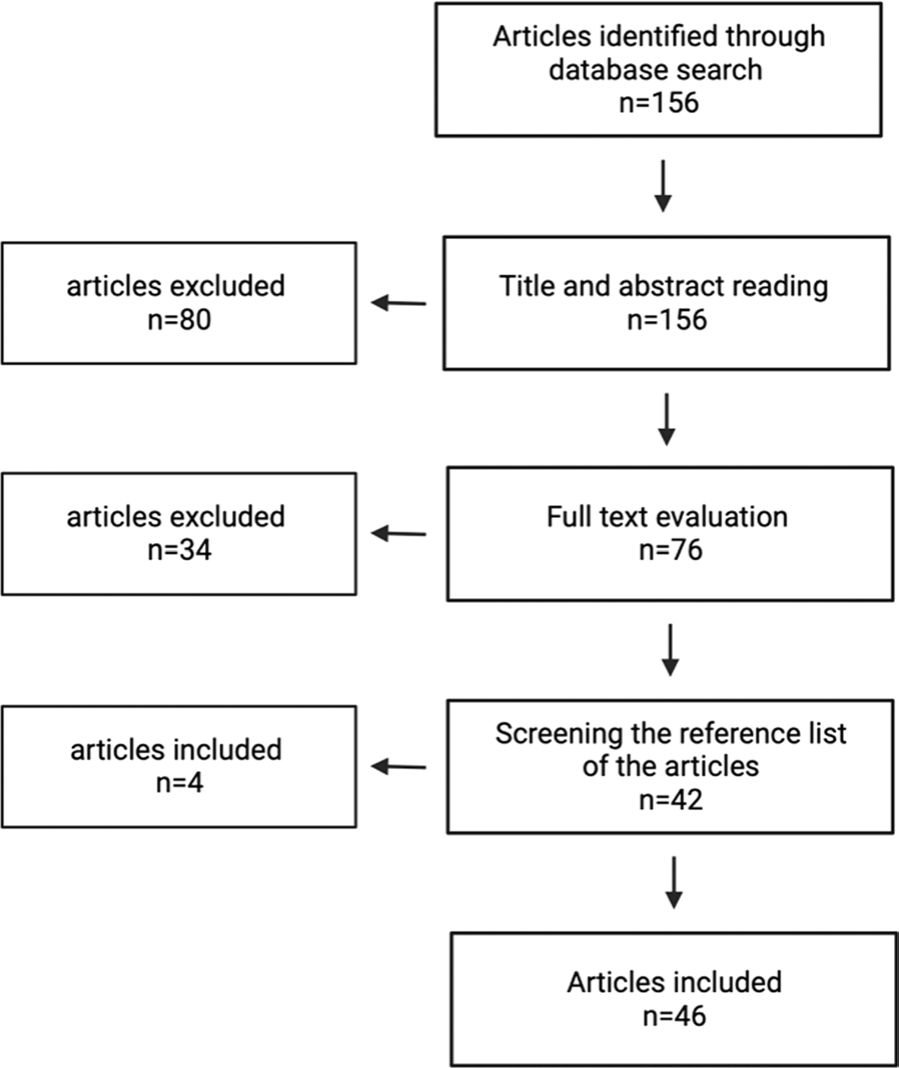
Summary of the literature search and selection process

The relevance of the publications was assessed and included following a defined flowchart (Fig. 1). First, inclusion was based on title and abstract reading. Selected articles were then accessed in full text to determine eligibility and extract the data. The reference lists of eligible publications were screened for additional articles. The scientific publications had to meet all the following criteria for inclusion: reported data had to include the number of VRE isolates and/or cases, and studies had to be conducted in a hospital. The following data were extracted from the selected publications: the first author’s name, country of origin, province of where the study was conducted, time frame for conducting the study, study methodology (outbreak report, surveillance report, prevalence/incidence study), hospital type, ward/ICU type, number of cases/samples involved in the study, the number and prevalence, incidence or proportion of VRE, and presence of resistance genes when available.

In addition, the national surveillance data from the two countries were reviewed by extracting information from NethMap in the Netherlands and the National Antimicrobial Resistance Surveillance (ARS) database established by the Robert Koch-Institute (RKI) in Germany and for both countries from EARS-Net data [31–33].

## Results

### Study inclusion and characteristics

**Fig. 2:**
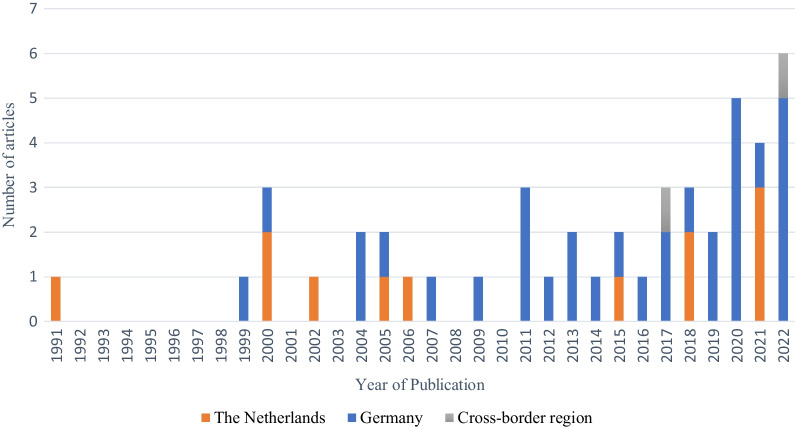
Publication dates of the included articles

The initial search yielded 156 potentially relevant publications, 80 of which were excluded based on title and abstract reading (Fig. 1). A further 32 publications were excluded after full-text evaluation. The reference lists of the eligible studies were screened, and four additional  studies were included. Ultimately, 46 publications were included (Figs. 1, 2). Of the selected publications, 12 were conducted in the Netherlands, and 32 in Germany. Two further studies were cross-border studies that included data from both countries. In total, there were one ecological, one pre-post study, one longitudinal study, four cohort studies, 14 outbreak reports, and 25 cross-sectional studies.

### Outbreaks due to vancomycin-resistant *E. faecium* (VREfm)

Of the 12 studies conducted in the Netherlands, eight were outbreak reports (Table 1) [34–41]. Of the 32 German publications, six were outbreak reports (Table 2) [42–47]. All outbreaks in both countries were caused by VREfm. In three of eight outbreaks observed in Dutch hospitals and in four of six outbreaks observed in German hospitals, VREfm infections were reported alongside patients colonized with VREfm [34, 37, 40, 42, 45–47]. One common factor observed in these reports was that colonization played a pivotal role in the occurrence of outbreaks in both countries.

**Table 1:** Summary of the studies on VRE carried out in Dutch hospitals (1991–2021)

Study	Design	Year	Setting	Patient population	Sample site	Clinical relevance	Species	Sample size	Outcome	Resistance gene (%)
*Outbreak Reports*										
Timmers et al*.* [34]	Outbreak report	1999	1 university hospital	Hematology ward	Anal, BSI	Infection, colonization	*E. faecium*	287 isolates	VRE isolates: 76 patients: 24 (2 infections) prevalence: 26.4%	*vanA* (100%)
Van der Steen et al*.* [35]	Outbreak report	2000	1 non-university hospital	Nephrology ward	Rectal, fecal, urine	Colonization	*E. faecium*	91 patients	Patients:8 prevalence: 19.8%	ND
Mascini et al*.* [36]	Outbreak report	2000–2003	1 university hospital	ICU, wards	Rectal	Colonization	*E. faecium*	183 patients	Patients:27 prevalence: 14.8%	ND
Frakking et al*.* [37]	Outbreak report	2012–2014	1 teaching hospital	ICU, wards	Rectal, BSI	Infection, colonization	*E. faecium*	ND	Patients: 242 (22 infections) prevalence: 4.3%	*vanA* (76%), *vanB* (13%)
Zhou et al. [39]	Outbreak report	2014	1 university hospital	Wards	Rectal, fecal, sputum, bile	Colonization	*E. faecium*	ND	VRE isolates: 36 patients: 34	*vanB* (94%), *vanA* + *vanB* (4%)
Weterings et al*.* [38]	Outbreak report	2014–2017	1 general hospital	ND	Rectal	Colonization	*E. faecium*	158 patients	Patients: 13 prevalence: 8%	ND
Lisotto et al*.* [40]	Outbreak report	2014, 2017	1 university hospital	Wards	Rectal, fecal, bile, pus, BSI	Infection, colonization	*E. faecium*	ND	VRE isolates: 39 (3 infections)	*vanB* (100%)
Gast et al*.* [41]	Outbreak report	2018	1 teaching hospital	ICU, oncology ward	Rectal, urine	Colonization	*E. faecium*	ND	Patients: 19	*vanB* (100%)
*Studies reporting on the prevalence of VRE colonization*										
Guiot et al*.* [49]	Cross-sectional	1991	1 university hospital	Hematology ward	Fecal	Colonization	*E. faecium, E. faecalis*	70 patients	Patients: 9 prevalence: 12.9%	ND
Van den Braak et al*.* [50]	Cross-sectional	1995–1998	5 university, 4 regional teaching hospitals	ICU, hematology-oncology ward	Rectal, fecal	Colonization	*E. faecium, E. faecalis*	1112 patients	Patients: 15 (*E faecium*, 11, *E. faecalis*, 4) prevalence: 1.3%	ND
Nys et al*.* [48]	Cohort	1999–2002	3 university hospital	Surgical wards	Fecal	Colonization	*E. faecalis*	261 patients	Patients: 3 prevalence: 1.1%	ND
*Studies reporting the frequency of VRE among all clinical and screening cultures*										
Aardema et al*.* [69]	Cross-sectional	2009–2010	1 university hospital	ICU	ND	Infection, colonization	ND	962 patients	Patients: 3 prevalence: 0.3%	ND

VRE isolates: number of detected isolates of VRE, patients: number of patients colonized/infected with VRE

*ICU:* intensive care unit, *ND:* not determined, *VRE:* vancomycin-resistant enterococci

**Table 2:** Summary of the studies on VRE carried out in German hospitals and hospitals in the Dutch-German cross-border region (1999–2022)

Study	Design	Year	Setting	Patient population	Sample site	Clinical relevance	Species	Sample size	Outcome	Resistance gene (%)
*Outbreak Reports*										
Elsner et al*.* [42]	Outbreak report	1993–1997	1 university hospital	Pediatric ICU/wards	ND	Infection, colonization	*E. faecium*	ND	Patients: 32 (5 infections)	*vanA* (100%)
Knoll et al. [43]	Outbreak report	1999–2001	1 university hospital	Hematology	Urine, fecal, axilla	Colonization	*E. faecium*	1124 patients	Patients: 44 prevalence: 3.9%	*vanA* (100%)
Borgmann et al*.* [44]	Outbreak report	2001	1 university hospital	NICU	Fecal	Colonization	*E. faecium*	ND	Patients: 24	*vanA* (100%)
Borgmann et al*.* [45]	Outbreak report	2004–2005	1 university hospital	ICU, wards	Rectal, fecal, wound, organ swabs	Infection, colonization	*E. faecium*	ND	Patients: 248 (94 infections)	*vanA* (90%)
Liese et al*.* [46]	Outbreak report	2010–2016	1 university hospital	All hospital	Rectal, fecal, intraoperative samples, ascites, aspirates, BSI	Infection, colonization	*E. faecium*	ND	VRE isolates*: 773 patients: 796 (159 infections)	*vanB (78.5%), vanA* (21.5%)
Bender et al*.* [47]	Outbreak report	2015–2019	2 hospitals	ND	Rectal, clinical specimen	Infection, colonization	*E. faecium*	ND	Patients: 2905 (127 infections)	*vanB* (98%), *vanA* (2%)
*Studies reporting on the prevalence of VRE colonization*										
Wendt et al*.* [51]	Cross-sectional	1995	1 university, 1 community hospital	ICU, surgical-medical wards	Rectal	Colonization	*E. faecium, E. faecalis*	552 isolates	Prevalence: 8.63% (university h), 1.77% (community h)	*vanA* (80%), *vanB* (20%)
Gruber et al*.* [52]	Cross-sectional	2006–2007	1 non-university hospital	Geriatric clinic	Rectal	Colonization	*E. faecium*	46 patients	Patients: 7 prevalence: 15.2%	ND
Liss et al*.* [53]	Cross-sectional	2008–2009	1 university hospital	Hematology-oncology	Fecal	Colonization	ND	513 patients	Patients: 51 prevalence: 9.9%	ND
Messler et al*.* [61]	Pre-post	2012–2013	1 university hospital	Surgical ICU	Rectal, clinical specimen	Colonization	*E. faecium*	2485 patients	Patients: 86. prevalence: 3.6%	*vanA* (61%), *vanB* (39%)
Neumann et al*.* [54]	Cohort	2014–2015	1 tertiary care hospital	Hematology-oncology	Rectal	Colonization	*E. faecium*	1606 patients	Patients: 111 prevalence: 23.8%	*vanB* (91%), *vanA* (9%)
Bui et al*.* [55]	Cross-sectional	2014–2015	1 university hospital	Wards (exc. ICU)	Rectal	Colonization	*E. faecium*	4013 patients	Patients: 48. prevalence: 1.2%	ND
Xanthopoulou et al*.* [56]	Cross-sectional	2014–2018	6 university hospitals	Wards (exc. ICU)	Rectal	Colonization	*E. faecium*	16,350 patients	Patients: 263; prevalence: 2014, 0.8%; 2015, 1.2%; 2016, 1.3%; 2017, 1.5%; 2018, 2.6%	*vanB (78.5%), vanA (20.2%), vanA* + *vanB (1.2%)*
Biehl et al*.* [62]	Cohort	2016	4 university hospitals	Hematology-oncology wards	Rectal, fecal	Colonization	*E. faecium, E. faecalis*	2928 patients	Patients: 176 (*E. faecium*, 173; *E faecalis,* 3). prevalence: 6%	*vanB* (77.8%), *vanA* (22%) *vanA* + *vanB* (0.2%)
Sommer et al*.* [57]	Cross-sectional	2017–2018	25 hospitals	All hospital	Rectal, wound	Colonization	*E. faecium*	629 patients	Prevalence: 5.7%	ND
Heininger et al*.* [58]	Cross-sectional	2018	1 university hospital	High risk patients at admission	Rectal	Colonization	*E. faecium*	2572 patients	Patients: 712 prevalence: 27.7%	ND
Chhatwal et al*.* [59]	Cross-sectional	2018–2019	1 university hospital	Hematology, oncology wards	Rectal, anal, fecal	Colonization	*E. faecium*	555 patients	Patients: 132 prevalence: 23.8%	*vanB* (93%), *vanA* (7%)
Trautmannsberger et al*.* [60]	Cross-sectional	2019–2020	1 university children’s hospital	NICU, PICU, surgical-medical wards	Rectal	Colonization	*E. faecium*	693 patients	Patients: 33 prevalence: 4.8%	*vanB* (54.5%), vanA (45.5%)
*Studies reporting the proportion of VRE in nosocomial infections and the incidence of VREfm in BSIs*										
Gastmeier et al*.* [67]	Cross-sectional	2007–2012	ICU-KISS, OP-KISS, Pathogen-KISS	ICU, surgical wards	Rectal, BSI, SSI UTI	Infection, colonization	*E. faecium E. faecalis*	ND	Nosocomial VRE infections: 2007–08, 79; 2009–10, 106; 2011–12, 14 proportion of VRE from 2007 to 2012: in SSI, 0.87% to 4.58%; in BSI, 4.91% to 12.99%; in UTI, 2.23% to 6.19%	ND
Remschmidt et al*.* [65]	Cross-sectional	2007–2016	ICU-KISS, OP-KISS	ICU (857), surgical wards (1119)	BSI, SSI, UTI	Infection	*E. faecium, E. faecalis*	ND	VRE infections: 2007–08, 79; 2009–10, 106; 2011–12, 143; 2013–14, 187; 2014–15, 318 proportion of VRE from 2007/2008 to 2015/2016: overall, 1.4% to 10%; in BSI, 5.9% to 16.7%; in UTI, 2.9% to 9.9%; in SSI, 0.9% to 5%	ND
Correa-Martinez, et al.[66]	Longitudinal	2016–2019	31 microbiology laboratories	ND	BSI	Infection	*E. faecium*	ND	VRE isolates: 755 incidence per 100,000 inhabitants: 2016, 0.48; 2019, 1.48	2016, *vanA* (64.5%); 2017, *vanB* (68.8%); 2018, *vanB* (83.1%); 2019, *vanB* (74.7)
Brinkwirth et al*.* [68]	Cross-sectional	2015–2020	ARS	ND	BSI	Infection	*E. faecium*	ND	VRE isolates: 3417 incidence per 100,000 inhabitants: 2015, 1.4%; 2020, 29%	ND
*Studies reporting the frequency of VRE among all clinical and screening cultures*										
Jones et al*.* [70]	Cross-sectional (surveillance)	2000–2002	169 hospitals	ICU	ND	Infection, colonization	*E. faecium E. faecalis*	621,636 isolates	Proportion of *E. faecium,* 4.8; proportion of *E. faecalis, 0.3*	ND
Remschmidt et al*.* [75]	Cohort	2001–2015	SARI (44 hospitals)	ICU (77)	ND	Infection, colonization	*E. faecium, E. faecalis*	263,639 isolates	ND	ND
Kohlenberg et al*.* [71]	Cross-sectional	2005–2006	MDR-KISS ICU	ICU (176)	Rectal, clinical specimen	Infection, colonization	*E. faecium, E. faecalis*	284,142 patients	Patients: 301 incidence per 1000 patient days: 0.1	ND
Scharlach et al*.* [79]	Cross-sectional	2006–2010	ARMIN—9 laboratories in Lower Saxony	ND	ND	Infection, colonization	*E. faecium*	6,672,431 isolates	VRE isolates: 2006, 667; 2010, 2431 proportion of VRE: 2006, 13.6; 2010, 5.6%	ND
Meyer et al*.* [74]	Cross-sectional	2007–2009	4 university hospitals	All hospital	ND	Infection, colonization	*E. faecium*	896,822 patients	Patients: 2007, 159; 2008, 277; 2009, 423 incidence per 10.000 patients: 2007, 5; 2008, 9; 2009,14)	ND
Kramer et al*.* [76]	Cross-sectional (point prevalence survey)	2010	5 tertiary, 4 secondary care hospitals	ICU, surgical-medical wards	ND	Infection, colonization	*E. faecium, E. faecalis*	3411 patients	Patients: 12 prevalence: 0.49%	ND
Huebner et al*.* [73]	Cross-sectional (point prevalence survey)	2012	37 acute-care hospitals	ICU, surgical-medical wards	ND	Infection, colonization	*E. faecium*	7154 patients	Prevalence: 0.38%	ND
Wegner et al*.* [77]	Cross-sectional (point prevalence survey)	2012	10 tertiary, 20 secondary, 26 primary care hospitals	ICU, surgical-medical wards	ND	Infection, colonization	*E. faecium, E. faecalis*	12,968 patients	Prevalence: 0.27%	ND
Huebner et al*.* [78]	Cross-sectional (point prevalence survey)	2014	45 tertiary, 76 secondary, 208 primary care hospitals	ICU, surgical-medical wards	ND	Infection, colonization	*E. faecium, E. faecalis*	73,938 patients/isolates	VRE isolates: 207 prevalence, 0.25%	ND
Remschmidt et al*.* [72]	Ecologic	2014–2015	1 university hospital	ICU, surgical-medical and hematology-oncology wards	Rectal, clinical specimen	Infection, colonization	*E. faecium, E. faecalis*	204,054 patients	Patients (n): 1430 prevalence: 0.7%	ND

VRE isolates: number of detected isolates of VRE, patients: number of patients diagnosed with VRE. *available

*ARMIN:* Antimicrobial Resistance Monitoring in Lower Saxony, BSI: blood-stream infection, *ICU:* intensive care unit, *KISS:* Krankenhaus-Infektions-Surveillance System (German national nosocomial surveillance system), *ND:* not determined, *NICU:* neonatal intensive care unit, *OP-KISS:* data on surgical site infections, *PICU:* pediatric intensive care unit, *SARI:* the surveillance of antibiotic use and resistance in intensive care units, *UTI:* urinary tract infection, SSI: surgical site infection, *VRE:* vancomycin-resistant enterococci

### Summary on the epidemiology of VRE

Thirty-two studies reported prevalence or incidence of VRE among inpatients: 17 studies reported the prevalence of VRE colonization, three studies reported the proportion of VRE in nosocomial infections, 11 studies reported the frequency of VRE among all clinical and screening cultures, and one study reported both the proportion of VRE in nosocomial infections and the frequency of VRE among clinical and screening cultures. Table 1, Table 2 and Table 3, which provide detailed epidemiological data, indicate whether the numbers presented correspond to VRE isolates or to the total number of patients diagnosed with VRE.

**Table 3:** Summary of the studies on VRE carried out in hospitals in the Dutch-German cross-border region (2012–2018)

Study	Design	Year	Setting	Patient population	Sample site	Clinical relevance	Species	Sample size	Outcome	Resistance gene (%)
*Studies reporting on the prevalence of VRE colonization*										
Zhou et al*.* [63]	Cross-sectional	2012–2013	2 university hospitals	ICU, wards	Rectal	Colonization	ND	NL: 445, DE: 102 isolates	VRE isolates: NL, 6; DE, 4 prevalence: NL, 1.3%; DE, 3.9%	NL: *vanB* (100%), DE: *vanB* (75%)
Glasner et al*.* [64]	Cross-sectional	2017–2018	8 Dutch, 15 German hospitals	ICU	Rectal	Colonization	*E. faecium*	NL: 1110, DE: 2035 isolates	VRE isolates: NL, 1; DE, 55 prevalence: NL, 0.1%; DE, 2.7%	ND

VRE isolates: number of detected isolates of VRE, patients: number of patients diagnosed with VRE

*ICU:* intensive care unit, *ND:* not determined, *DE:* Germany, *NL:* the Netherlands

#### Studies reporting on the prevalence of VRE colonization

Of the 17 studies that reported on the prevalence of VRE colonization, three were from Dutch hospitals and 14 were from German hospitals. One cohort study and two cross-sectional studies investigated VRE colonization in different patient groups in Dutch hospitals (Table 1). In the cohort study, the prevalence of vancomycin-resistant *E. faecalis* colonization was 1.1% in surgical patients from three university hospitals [48]. In the cross-sectional studies, the prevalence of VRE colonization (*E. faecalis* and *E. faecium)* was 12.9% in the study conducted in hematology patients of the university hospital in Leiden in 1991 [49] and 1.3% in the study involving intensive care and hematology-oncology patients from nine different hospitals between 1995 and 1998 [50].

The prevalence of VRE colonization in different patient groups was investigated in nine cross-sectional studies, two cohort studies and one pre-post study in German hospitals. The prevalence ranged between 1.2% and 27.7% (Table 2) [51–62]. All studies reported the prevalence of VREfm colonization, except for three studies, one that did not specify the species and the other two that reported both *E. faecalis* and *E. faecium* [51–62]. The highest prevalence was reported in studies among hematology-oncology patients (23.8%), geriatric patients (15.2%), and patients at high risk (27.7%) for VREfm colonization [52, 54, 58, 59]. The lowest VREfm colonization (1.2% and 1.6%) prevalence was reported in two hospital-wide studies, which did not include intensive care unit (ICU) patients [55, 56]. One of these studies was carried out in six university hospitals throughout Germany and found an increase in VREfm colonization prevalence (0.8% in 2014, 1.2% in 2015, 1.3% in 2016, 1.5% in 2017, 2.6% in 2018) in inpatients over the years between 2014 to 2018 [56].

Two cross-border studies compared the prevalence of VRE colonization among hospitalized patients (Table 3). In one of the studies conducted at two university hospitals in the Northern Dutch-German cross-border region between 2012 and 2013, the prevalence of VRE colonization in the German hospital (3.9%) was three times higher than in the Dutch hospital (1.3%) [63]. The difference was even more significant in the study carried out in 23 hospitals’ ICUs (8 Dutch and 15 German) in the cross-border region between 2017 and 2018: VRE colonization prevalence was almost 30 times higher in the German hospitals (2.7%) than in the Dutch hospitals (0.1%) [64].

#### Studies reporting the proportion of VRE in nosocomial infections and the incidence of VREfm in bloodstream infections (BSIs)

The studies that reported the proportion of nosocomial, invasive VRE were all conducted in German hospitals and presented an increase in VRE infections in Germany over the years (Table 2) [65–68]. Two studies analyzed data from the German National Nosocomial Surveillance System (KISS, Krankenhaus-Infektions-Surveillance-System, https://www.nrz-hygiene.de/kiss/kiss-module) and reported the proportion of VRE (*E. faecium* and *E. faecalis)* in nosocomial infections. The first study analyzed the proportion of VRE in nosocomial infections in ICUs and surgical departments between 2007 and 2012 [67]. This study found not only an increasing trend of VRE (from 2007 to 2012: in SSI, 0.87% to 4.58%; in BSI, 4.91% to 12.99%; in UTI, 2.23% to 6.19%) in Germany in general, but also a diversity between federal states including a “VRE belt” in the middle of the country, ranging from the West (North Rhine-Westphalia) to East (Saxony) [67]. The second study described a continuous increase in nosocomial infections caused by VRE in German ICUs and surgical wards from 1.4% in 2007 to 10% in 2016 [65].

The remaining two studies reported the incidence density of VREfm in bloodstream infections (BSI). The first study was a prospective longitudinal study in 31 laboratories in North Rhine-Westphalia, Germany [66]. This study found an increase in the incidence density (per 100,000 inhabitants) of VREfm BSI from 0.52 in 2016 to 1.48 in 2019 [66]. The second study  analyzed the ARS surveillance system, which reported an increasing estimated incidence density (per 100,000 inhabitants) of VREfm BSI from 1.4 in 2015 to 2.9 in 2020 across the country [68].

#### Studies reporting the frequency of VRE among all clinical and screening cultures

All 12 studies (one conducted in a Dutch hospital and 11 in German hospitals) analyzed microbiology data without distinguishing between VRE infection or VRE colonization. Unless otherwise stated, the reported numbers represent the combined rate of VRE in both *E. faecium* and *E. faecalis* isolates. The study conducted at the Dutch hospital (University Hospital Groningen) was a cross-sectional study, reporting a prevalence of 0.3% VRE in ICU patients [69].

Of the 11 German studies, nine were cross-sectional studies, one was a cohort study, and one was an ecologic study (investigating the impact of antibiotic use on VRE prevalence). An international surveillance study, including data from 169 German hospitals between 2000 and 2002, reported a VRE prevalence of 4.8% for *E. faecium* and 0.3% for *E. faecalis* [70] and a study that analyzed MDR-KISS data between 2005 and 2006 reported a VRE prevalence of 0.1% in ICU patients [71]. The ecologic study that was conducted at the university hospital Berlin in 2012 reported a VRE prevalence of 0.7% [72]; in a point prevalence study conducted in 37 acute-care hospitals in Munich in 2012 a VREfm prevalence of 0.38% was recorded in inpatients, including ICU patients [73].

Three of the cross-sectional studies reported an increasing incidence of VRE over several years. In one of the studies that was conducted at four university hospitals across different regions in Germany (East, North, Southwest, Southeast), an increase in the incidence (with rates rising from 5 to 9 to 14 per 10,000 patients) of VREfm was observed between 2007 and 2009 [74]. Two studies that analyzed the data from KISS and the Surveillance of Antibiotic use and Resistance in ICUs (SARI) project also recorded an increase in VRE in German hospitals [67, 75]. The incidence of VRE cases (per 100 admitted patients) in ICUs rose from 0.11 in 2007 to 0.31 in 2012 [67], whereas the resistance density of VRE in German ICUs increased from 0.1 in 2001 to 1.1 per 1000 patient days in 2015 in the other study, which included the SARI cohort [75]. In contrast, three nationwide one-day point prevalence studies conducted in 2010, 2012, and 2014 using the same study protocol but with different numbers of participating hospitals did not show an increase in VRE colonization or infection among hospitalized patients [76–78]. In addition to these national studies, a regional study was conducted to identify regional trends of AMR in Lower Saxony. In this study, the data of the Antimicrobial Resistance Monitoring in Lower Saxony (ARMIN) project in the period 2006–2010 were analyzed, and strikingly, this study reported a decreasing proportion of VREfm cases within those years in Lower Saxony from 13.6% in 2006 to 5.6% in 2010 [79].

### Molecular epidemiology of VRE over time

Data from outbreaks in both Dutch and German hospitals revealed that the molecular epidemiology of VREfm causing outbreaks has changed from a predominance of *vanA* towards *vanB* over the years (Table 1–2) [34, 37, 39–47].

In the Netherlands, of the eight reported outbreaks, six reported the *vanA/B* status of isolates. In an outbreak in 1999 at the university hospital in Amsterdam, all VREfm isolates were *vanA*-positive [34], and in another outbreak at a non-university hospital in Utrecht between 2012 and 2014, the majority of the VREfm isolates were *vanA*-positive [37]. In contrast, two outbreaks at the university hospital in Groningen in 2014 and 2017 were predominantly caused by *vanB*-VREfm [39, 40]. Similarly, in an outbreak at a tertiary hospital in Tilburg in 2018, all VREfm isolates were *vanB*-positive [41].

In Germany, all reported VREfm outbreaks provided molecular data. The outbreaks at the university pediatric hospital in Hamburg (1993–1997), at the university hospital in Halle (1999–2001), and at the university hospital in Tübingen (2001) were all caused solely by *vanA* VRE [42–44]. In another outbreak at the university hospital in Tübingen in 2004, most VREfm isolates were *vanA-*positive [45]. In a hospital-wide outbreak at a university hospital in south-west Germany in 2015 [46] and in a VREfm outbreak in two regional hospitals in southern Germany between 2015 and 2019, *vanB* was most frequently detected [47].

Apart from the above-mentioned outbreak reports, no other studies from the Netherlands reported molecular data of VRE. However, a shift from *vanA* to *vanB* over time was also observed in German non-outbreak studies (Table 2). In a cross-sectional study at two hospitals in Berlin in 1995 [51] and another at the university hospital in Cologne between 2012 and 2013, most isolates were *vanA*-positive [61]. In contrast, most studies after 2013 reported a predominance of *vanB*, including a study at a tertiary care hospital in southern Germany (2014–2015) [54], a cohort study at the university hospitals in Cologne, Freiburg, Hamburg, and Tübingen (2016) [62], and a cross-sectional study at six university hospitals throughout Germany (2014–2018) [56]. In a longitudinal study in 31 microbiology laboratories in North Rhine-Westphalia, *vanA* was predominant in 2016, while *vanB* was most prevalent in 2017–2019 in VRE BSIs [66]. Similarly, in a study in 2018–2019 at the university hospital in Hannover [59] and another study in 2019 in Munich [60], *vanB* was more frequent than *vanA*.

### VRE surveillance data reports on the national level

Both countries have their own national antibiotic resistance surveillance systems, including VREfm, and both submit their results to EARS-Net.

#### The Netherlands

**Fig. 3:**
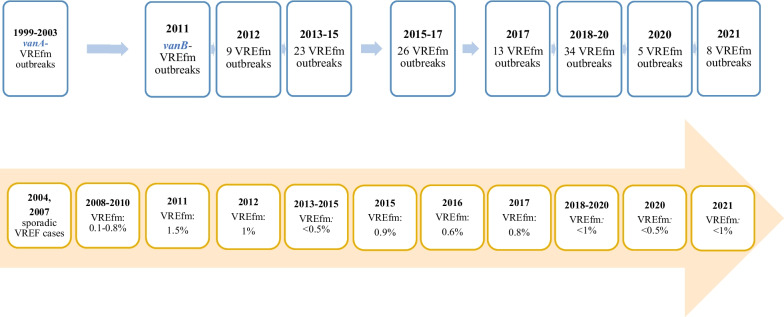
Summary of the number of VRE*fm* outbreaks (blue boxes) and VREfm proportion (orange boxes) in clinical isolates in Dutch hospitals between 2003 and 2021 (NethMap reports) [81]. The data in the boxes represent the temporal distribution of VRE data over the years. (*VREF: vancomycin resistant E. faecalis, VREfm: vancomycin- resistant E. faecium*)

Microbiological data of all isolates from medical microbiology laboratories in the Netherlands are collected in the Infectious Diseases Surveillance Information System for Antimicrobial Resistance (ISIS-AR) [80]. Based on these data and in collaboration with the Dutch Working Group on Antibiotic Policy of the Dutch Society of Medical Microbiology, a SWAB/RIVM report (NethMap) has been published annually to monitor AMR since 2003 [81]. Data regarding VRE from clinical isolates have been available since 2003 in NethMap reports (Fig. 3).

According to the NethMap reports, there was a significant increase (from 0.1–0.8% to 1.5%) in the proportion of *vanB*-positive VREfm in hospitals between 2008 and 2011. This increase was attributed to VREfm outbreaks, particularly occurring in hospitals in the northern region of the country [81]. As Fig. 3 shows, numerous VREfm outbreaks have been reported in the Netherlands over the years. However, the proportion of VREfm in clinical isolates of *E. faecium* in hospitals remained below 1% and has not changed in the last decade. To manage and prevent large-scale outbreaks of AMR in healthcare facilities and contain its spread to other institutions at an early stage, the Early Warning and Intervention Meeting for Nosocomial Infections and Antimicrobial Resistance (SO-ZI/AMR), was established in the Netherlands in 2012 [82]. Participating hospitals have voluntarily committed to the SO-ZI/AMR system, which includes reporting obligations and regular updates until the outbreak is resolved. Of all VREfm outbreaks in the last decade, the lowest numbers were recorded in 2020 and 2021. This decrease could potentially be influenced by multiple factors such as the implementation of enhanced infection control measures during the COVID-19 pandemic or a potential decrease in reporting due to the burden of the pandemic, as reporting is voluntary.

There is currently no nationwide surveillance of the molecular epidemiology of VRE in the Netherlands. Centrally collected national data on VREfm molecular typing were available only between 2012 and 2018, and *vanA* was always more frequent than *vanB* during this period [81].

#### Germany

**Fig. 4:**
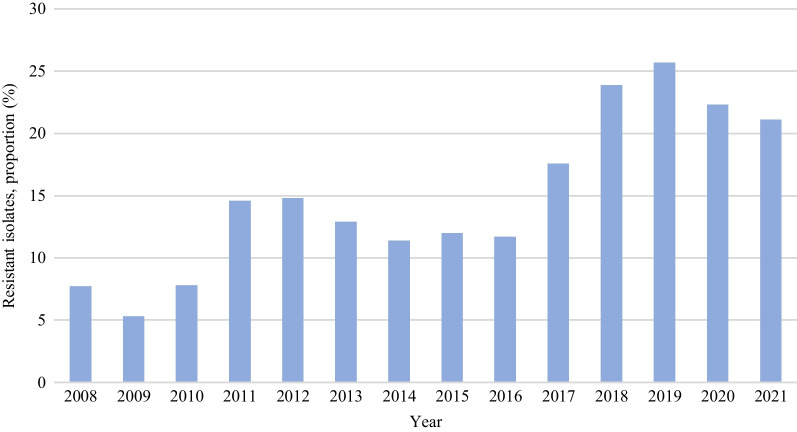
VRE*fm* as the proportion (%) of all *E. faecium* isolates from inpatients’ blood cultures between 2008 and 2021 in Germany (ARS-RKI Statistics) [85].* (VREfm: vancomycin- resistant E. faecium*)

Microbiological data of all isolates from participating medical microbiology laboratories and hospitals in Germany are collected in the ARS database established by the RKI since 2008 [33]. Pre-2008 national data are available in so-called Epidemiology Bulletins, which have been periodically published by the RKI. According to these reports, there was an increase in the number of VREfm isolates observed in 2003 and 2004 (both screening and clinical samples) compared to the previous years [83]. Following a short decrease in the following two years, numbers increased again in 2007 [84]. The ARS database, available since 2008, provides data regarding the proportion (%) of VREfm in all *E. faecium* isolates obtained from inpatient blood cultures (Fig. 4). Since 2009 an overall increasing trend of the VREfm proportion could be observed.

A National Reference Center (NRC) for staphylococci and enterococci was assigned by RKI in 2012 [86]. According to the NRC, significantly more *vanB*-VREfm than *vanA*-VREfm isolates were sent to the NRC for the first time in 2017, and the situation has remained the same since then [87, 88].

#### EARS-net

**Fig. 5:**
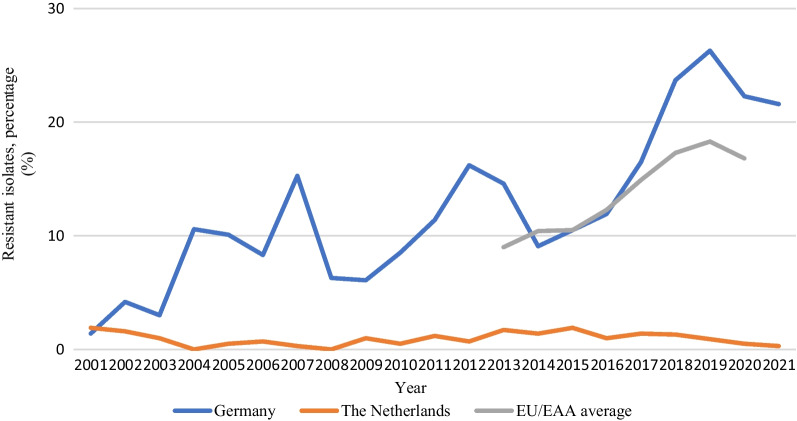
The percentage of VREfm in clinical (invasive) *E. faecium* isolates in the Netherlands and Germany between 2001 and 2021. EU/EAA average was only reported between 2013 and 2020. Data from the ECDC Surveillance Atlas [92]

The national AMR data represented in EARS-Net are obtained from the RIVM and RKI in the Netherlands and Germany, respectively [24]. In 2021, the population coverage in the EARS-Net surveillance data was 68% for the Netherlands and 35% for Germany [89]. Throughout the years, the coverage percentages have remained relatively stable, with the Netherlands consistently having higher coverage compared to Germany [90]. The Netherlands is among 13 out of 30 countries that have maintained a VRE rate below 5% in clinical *E. faecium* isolates over the course of several years. In contrast, in Germany, the percentage increased continuously between 2016 (11.9%) and 2019 (26.3%) and surpassed the European average since 2017 (Fig. 5) [24]. Interestingly, this percentage (22.3%) decreased in 2020 for the first time since 2014 [91].

## Discussion

Given the limited treatment options and increasing prevalence of VRE in Europe, VRE remains a severe problem in healthcare [5, 24]. Despite this overall increase, large variations have been reported between countries [24]. To the best of our knowledge, we provide the first comparative overview of the epidemiology of VRE in hospital settings in the Netherlands and Germany, covering 102 million EU inhabitants, by reviewing the literature and national surveillance data.

In this review, the studies from the two countries did not only differ in number but also in the type of design. While most of the studies in the Netherlands were outbreak reports, cross-sectional prevalence studies were predominant in Germany. The larger number of cross-sectional prevalence studies in German hospitals may indicate that VRE is a more pertinent problem in German than in Dutch hospitals.

Analysis of outbreak reports revealed that all outbreaks in both countries were caused by VREfm. This is not surprising because of the high tenacity of *E. faecium* to survive in the hospital environment [93]. Although the rate of infections differed within and between countries, colonization was a common cause of VREfm outbreaks in both countries. Studies on prevalence or incidence of VRE varied considerably depending on the patient population and time. Generally, high VRE prevalences were reported in high-risk wards such as haemato-oncology and geriatric wards in both countries [49, 52–54, 59]. This finding is consistent with previous studies, which have identified age and haemato-oncological malignancies as risk factors for both VRE colonization and infection [94–96].

The most prominent difference between the two countries was that the German studies showed an increasing trend of VRE prevalence in German hospitals, yet such a trend was not observed in the Dutch studies. It is important to acknowledge that the smaller number of Dutch studies restricts the ability to draw conclusive observations regarding this matter. Cross-border studies have also demonstrated this difference when applying the same screening strategy for hospitalized patients [63, 64]. This observation is supported by the national data of both countries and EARS-Net data. EARS-Net data shows that the proportion of VREfm in clinical *E. faecium* isolates from patients with invasive infections has remained stable,  with slight fluctuations below 1% in the Netherlands over the past decade, while in Germany, it has risen to over 25% with an increasing trend [24, 65].

In the following paragraphs, we will elaborate on some points that may explain the difference in epidemiology of VRE between these two neighboring countries.

### Healthcare system

The inherent differences in healthcare structures could serve as a primary explanation for this difference [25, 28]. Both Germany and the Netherlands have well-established healthcare systems, however, they differ in important aspects [28]. Firstly, the density of inpatient care (number of cases), the average length of hospital stay, and bed occupancy rate were found to be significantly higher in Germany-all factors that could increase the risk of VRE transmission through increased patient-to patient and patient-to-healthcare professional (HCP) contact [28]. As the hospital environment is one of the key factors for VRE transmission via surfaces, a high occupancy rate in hospitals would also facilitate the spread of VRE [16]. In addition, high bed occupancy rates result in fewer single rooms available to isolate patients with VRE, making it challenging to implement adequate IPC rules in German hospitals [97]. In contrast, even pre-emptive isolation is implemented for at risk patients upon admission in Dutch hospitals [28, 98]. Secondly, despite the high number of hospitalizations and longer hospital stays, German hospitals suffer more compared to Dutch hospitals from a shortage of HCPs, resulting in understaffing, particularly in nursing care [28]. The interaction between patients and HCPs has a crucial role in VRE transmission, which may be one of the factors contributing to the high VRE prevalence in German hospitals, due to the low nurse-to-patient ratio [99].

### Infection control guidelines

In addition to the differences in healthcare structure, there are also variations in the national German and Dutch IPC guidelines for the prevention of VRE in hospitals [25]. The frequency of MDROs in hospitals could serve as an indicator of the effectiveness of IPC measures. In Germany, the Commission for Hospital Hygiene and Infection Prevention (KRINKO, Kommission für Krankenhaushygiene und Infektionsprävention), and in the Netherlands the Infection Prevention Working Group (WIP, Werkgroep Infectie Preventie, Samenwerkingsverband Richtlijnen Infectiepreventie), issue these national IPC guidelines [98, 100, 101]. In general, while the application of IPC rules in the German guideline varies according to the epidemiological situation of the hospital and region, there is no such exception in the Dutch guideline. The KRINKO guidelines primarily focus on prevention of infections requiring antibiotic therapy, classifying patient groups according to their risk of evolving VRE infection, whereas the WIP guidelines recommend a search and detect strategy. For instance, in the WIP guidelines, there is no distinction between high-risk wards and normal-care wards in VRE screening, whereas the KRINKO guidelines recommend VRE screening only on patients in high-risk wards. The management of VRE carriers also differs in the two guidelines; the WIP guidelines recommend contact isolation without exception, but the KRINKO guidelines leaves the decision to clinicians, based on the patient's risk assessment. Thus, the stricter infection control rules applied in Dutch hospitals could contribute to the lower prevalence of VRE.

### Antibiotic consumption

In addition to well-established IPC measures and the level of compliance with these measures, appropriate use of antibiotics plays a significant role in preventing colonization with VRE and, hence, infection [102]. For instance, the use of broad-spectrum cephalosporins has been linked to an increased VRE prevalence, both by facilitating the acquisition of VRE and by exerting high selective pressure on the gastrointestinal flora [103–106]. Data from the European Surveillance of Antimicrobial Consumption Network (ESAC-Net) from 1997 to 2020 indicate that the use of broad-spectrum cephalosporins in the community in Germany was higher than in the Netherlands [107]. Given this difference in the use of this particular antibiotic group between the two countries, it is possible that this will also have an impact on the difference in VRE prevalence observed between them.

### Diagnostics

Apart from the aforementioned differences that have been outlined between the Netherlands and Germany, it is important to consider that variations in the diagnostic laboratory protocols, guidelines, and availability of resources for detecting VRE may also play a role in influencing the reported VRE cases in each country [64]. Variations in diagnostic protocols, including sample collection, culturing techniques, and antimicrobial susceptibility testing, can impact VRE detection. For example, variances in media and selective agents used for VRE isolation affect sensitivity and specificity [108]. Differences in the adoption and implementation of surveillance guidelines can also affect VRE detection and reporting, particularly in screening frequency and extent for VRE colonization in specific patient populations [98, 100]. Additionally, the availability of resources (financial, technological, and human) plays a significant role in a laboratory's capacity to detect VRE, with advanced technologies like PCR assays improving sensitivity and speed [109]. These factors can potentially impact the accuracy and thoroughness of VRE detection and reporting, thus contributing to variations in the reported number of VRE cases between the two countries.

### Commonalities

Even though the general development in VRE epidemiology in the Netherlands and Germany differed substantially in the last decades, two common trends have emerged. The first trend is the potential impact of the COVID-19 pandemic on VRE epidemiology. Data from EARS-Net reports for 2020 and 2021 indicate that the number of VRE outbreaks and the proportion of VRE among all *E. faecium* isolates from clinical isolates have decreased in both countries compared to the previous year [110]. This decline could be due to an increased awareness of IPC measures among healthcare professionals and the disruption of healthcare services due to the COVID-19 pandemic. However, it is also possible that deprioritization of AMR surveillance in hospitals and less engagement to national surveillance systems may have led to an underestimation of actual situation.

The second trend is the change in the molecular epidemiology of VRE over time. In Germany, molecular typing analyses have been performed on all enterococci submitted to the NRC, while in the Netherlands, such analyses were only available for centrally collected enterococci between 2012 and 2018. Apart from the national surveillance data, identified publications illustrated that *vanB* began to be reported as the leading cluster both in the Netherlands and in Germany, since 2014 [39–41, 54, 56, 59]. This shift in molecular epidemiology has led to debate about whether this change is a result of an actual rise in the circulation of *vanB* strains or limitations in the detection of *vanB*-VRE in the laboratory [111]. Comparative studies have revealed that gradient strip assays and automated antibiotic susceptibility testing methods commonly used in the routine laboratory setting fail to detect *vanB*-mediated vancomycin resistance [112, 113]. EUCAST has also acknowledged these issues and revised recommendations to reduce the error rate in detecting *vanB-*VRE [114].

### Limitations

There are limitations to this study. Firstly, a meta-analysis was not possible due to the heterogeneity in study design, patient populations, timeframes, and outcome definitions across the publications. Secondly, comparing the national surveillance data might cause biases owing to the changing number of participating hospitals and laboratories and different data collection compliance in the two countries. Thirdly, a comprehensive comparison of implementation and compliance to the national IPC guidelines at the hospital level was beyond the scope of the current study, disallowing us to compare the real-life records of hospital practice.

## Conclusion

In conclusion, this review has provided an overview of the epidemiology of VRE in the hospital setting in the Netherlands and Germany, highlighting the potential causes for the difference in VRE prevalence between these neighboring countries. Given the increasing prevalence of VRE in Europe, we demonstrate that VRE remains a serious problem in healthcare and call for further research to understand the underlying factors driving the difference in VRE prevalence between countries to develop effective strategies to control the spread of VRE.

### Supplementary Information

**Additional file 1**. The final applied search term.

**Additional file 2**. Dataset presenting the extracted data.

## Data Availability

Not applicable.

## Abbreviations

AMR: Antimicrobial resistance
BSI: Blood-stream infection
ARMIN: Antimicrobial Resistance Monitoring in Lower Saxony
ARS: National Antimicrobial Resistance Surveillance
CHARE-GD: Comparison of healthcare structures, processes and outcomes in the Northern German and Dutch cross-border region
EARS-Net: European Antimicrobial Resistance Surveillance Network
ESAC-Net: European Surveillance of Antimicrobial Consumption Network
HCP: Healthcare professional
ICU: Intensive care unit
IPC: Infection prevention and control
ISIS-AR: Infectious Diseases Surveillance Information System for Antimicrobial Resistance
MDRO: Multidrug-resistant microorganism
NICU: Neonatal intensive care unit
KISS: Krankenhaus-Infektions-Surveillance-System (Hospital Infection Surveillance System from Germany)
KRINKO: Kommission für Krankenhaushygiene und Infektionsprävention (Commission for Hospital Hygiene and Infection Prevention in Germany)
PICU: Pediatric intensive care unit
RIVM: Rijksinstituut voor Volksgezondheid en Milieu (National Institute for Public Health and the Environment in the Netherlands)
RKI: Robert Koch Institute
SARI: Antibiotic use and Resistance in ICUs
SSI: Surgical site infection
SO-ZI/AMR: Signaleringsoverleg Zorginstellingen en Antimicrobiële Resistentie (The Early Warning and Intervention Meeting for Nosocomial Infections and Antimicrobial Resistance in the Netherlands)
UTI: Urinary tract infection
VRE: Vancomycin-resistant enterococci
VREfm: Vancomycin-resistant *E. faecium*
VSE: Vancomycin-susceptible enterococci
WIP: Werkgroep Infectie Preventie (Infection Prevention Working Group in the Netherlands)
WHO: World Health Organization
