# Supplementary material for: Vancomycin-resistant enterococci (VRE) in hospital settings across European borders: a scoping review comparing the epidemiology in the Netherlands and Germany
Source: Antimicrob Resist Infect Control. 2023 Aug 12;12:78. doi: 10.1186/s13756-023-01278-0 (PMC10422769; doi:10.1186/s13756-023-01278-0)
Supplement: Supplementary file 1 — Additional file 1. The final applied search term. [file 13756_2023_1278_MOESM1_ESM.docx]

The final applied search term.

*("Vancomycin-Resistant Enterococci"[Mesh] OR (("Enterococcus"[Mesh]) AND ("Vancomycin Resistance"[Mesh])) OR ((vancomycin[tiab]) AND (resistan*[tiab]) AND (enterococc*[tiab])) OR VRE[tiab]) AND ("Hospitals"[Mesh] OR hospital*[tiab] OR "Inpatients"[Mesh] OR inpatient*[tiab] OR "Intensive Care Units"[Mesh] OR ICU*[tiab] OR nosocomial[tiab] OR centre*[tiab] OR ward*[tiab]) AND ("Prevalence"[Mesh] OR prevalence*[tiab] OR outbreak*[tiab] OR surveillance*[tiab] OR "mass screening"[Mesh] OR screening*[tiab] OR colonization*[tiab] OR "Incidence"[Mesh] OR incidence[tiab] OR "Epidemiology"[Mesh] OR epidemiology[tiab] OR "Epidemics"[Mesh] OR epidemic*[tiab]) AND ("Netherlands"[Mesh] OR Netherland*[tiab] OR Dutch*[tiab] OR Holland[tiab] OR "Germany"[Mesh] OR German*[tiab]).*
